# Does Music Training Enhance Literacy Skills? A Meta-Analysis

**DOI:** 10.3389/fpsyg.2015.01777

**Published:** 2015-12-01

**Authors:** Reyna L. Gordon, Hilda M. Fehd, Bruce D. McCandliss

**Affiliations:** ^1^Music Cognition Lab, Program for Music, Mind and Society, Department of Otolaryngology, Vanderbilt University Medical CenterNashville, TN, USA; ^2^Vanderbilt Kennedy Center, Vanderbilt University Medical CenterNashville, TN, USA; ^3^Institute for Software Integrated Systems, School of Engineering, Vanderbilt UniversityNashville, TN, USA; ^4^Department of Psychology, Graduate School of Education, Stanford UniversityStanford, CA, USA

**Keywords:** music training, reading, literacy, phonological awareness, meta-analysis, brain development

## Abstract

Children's engagement in music practice is associated with enhancements in literacy-related language skills, as demonstrated by multiple reports of correlation across these two domains. Training studies have tested whether engaging in music training directly transfers benefit to children's literacy skill development. Results of such studies, however, are mixed. Interpretation of these mixed results is made more complex by the fact that a wide range of literacy-related outcome measures are used across these studies. Here, we address these challenges via a meta-analytic approach. A comprehensive literature review of peer-reviewed music training studies was built around key criteria needed to test the direct transfer hypothesis, including: (a) inclusion of music training vs. control groups; (b) inclusion of pre- vs. post-comparison measures, and (c) indication that reading instruction was held constant across groups. Thirteen studies were identified (*n* = 901). Two classes of outcome measures emerged with sufficient overlap to support meta-analysis: phonological awareness and reading fluency. Hours of training, age, and type of control intervention were examined as potential moderators. Results supported the hypothesis that music training leads to gains in phonological awareness skills. The effect isolated by contrasting gains in music training vs. gains in control was small relative to the large variance in these skills (*d* = 0.2). Interestingly, analyses revealed that transfer effects for rhyming skills tended to grow stronger with increased hours of training. In contrast, no significant aggregate transfer effect emerged for reading fluency measures, despite some studies reporting large training effects. The potential influence of other study design factors were considered, including intervention design, IQ, and SES. Results are discussed in the context of emerging findings that music training may enhance literacy development via changes in brain mechanisms that support both music and language cognition.

## Introduction

Acquiring fluency in reading requires children to transform symbolic information provided by print into mental representations based on their prior language experience. This literacy acquisition relies heavily on the process of phonological awareness. In particular, children's ability to focus their attention on sub-syllabic phonological units within words is a critical factor for mastering the early challenge of alphabetic decoding. Phonological awareness has also been linked to neural mechanisms that help explain individual differences in early literacy (Schlaggar and McCandliss, 2007). Moreover, a growing number of studies have linked music skills and music training to differences in speech perception (Wong et al., 2007; François and Schön, 2011); basic auditory perception (Shahin et al., 2003; Hyde et al., 2009) and acquisition of second language or an artificial language (Slevc and Miyake, 2006; Brod and Opitz, 2012). Basic auditory processing appears to be a building block of phonological awareness (Walker et al., 2006), and music training is associated with both superior auditory perception (Seither-Preisler et al., 2014) and enhanced language skills (see Patel, 2008, for a review).

Understanding the potential connection between music training and literacy skills is informed by two areas of research literature. The first is a well-established body of research showing that some language-related skills, such as phonological awareness, are a fundamental pre-cursor of reading skills (see meta-analysis by Melby-Lervag et al., 2012), and the second is an emerging literature investigating the potential role of music training as an activity that may induce plastic changes and perceptual enhancements within neural systems crucial for reading (e.g., Kraus et al., 2014b). Learning to play an instrument or to sing requires a complex series of neural transformations in order to process fine-grained acoustic variations in timing, frequency, spectral characteristics, and intensity into musically relevant auditory-motor actions to create rhythm, pitch, timbre, and dynamics. The OPERA hypothesis (Patel, 2011, 2014) provides a framework for highlighting the multiple perceptual demands musical training requires and the benefits such demands may bestow on neural systems that are important for literacy and language skills. Together, these two literatures provide constraints on understanding pathways through which musical training may enhance early literacy acquisition.

A rapidly accumulating body of evidence has shown associations between language and music skills in children. For instance, 7-to-9-year-old musicians outperformed their non-musician peers at detecting small prosodic (pitch) incongruities in sentences (Magne et al., 2006). Likewise, 9-year-olds musicians (vs. non-musicians) showed enhanced brain responses and behavioral performance on detection of deviants of the voice-onset-time, frequency, and duration of syllables (Chobert et al., 2011). Foreign language pronunciation skills and brain response to duration deviants (in music and speech) were better in 10- to 12-year-olds with musical training (Milovanov et al., 2009). Even without explicit music training, some of the variability in language skills can be accounted for by measuring individual differences in music aptitude. Measures of music aptitude have been found to account for over 40% of the variance in reading performance in typically developing 8- to 13-year-old children with little to no music training (Strait et al., 2011). Rhythm perception skills were robustly correlated with grammar production skills in 6-year-olds (Gordon et al., 2015b); a follow-up study of grammatical categories and musical rhythm revealed that musical rhythm explains production of complex sentence structure in particular (Gordon et al., 2015a).

Reading is one language skill that has received recent attention in the neuroscience community regarding potential shared neural resources with music. Anvari et al. (2002) showed that pitch and rhythm skills in 4- and 5-year-olds correlated with phonological awareness and early reading skills, converging with prior findings of a correlation between pitch discrimination and both phonemic awareness and early reading abilities in a similar age group (Lamb and Gregory, 1993). Musical rhythm in particular has been linked to reading skills in prior work using a wide variety of methods for measuring rhythm in young children, across many native languages. American-English-speaking preschoolers who excelled at synchronizing to an acoustic beat (“Synchronizers”) outperformed their “Non-synchronizer” peers at phonological awareness and rapid naming tasks (Woodruff Carr et al., 2014). A French study with large sample size (*n* = 695) showed that kindergarteners' ability to reproduce musical rhythms was significantly predictive of their second grade reading skills (Dellatolas et al., 2009). Interestingly, Banai and Ahissar (2013) found a stronger relationship between reading and auditory processing skills in Israeli children without musical training, while the musician children in the study showed better auditory processing but no advantage in reading skills.

The relation between rhythm and reading-related skills continues to be significant in later stages of language development. Tierney and Kraus (2013b) found that beat tapping variability (to an isochronous metronome at a 2 Hz rate) negatively correlated with reading skills in adolescents, such that those who tapped to the beat more consistently were more likely to have better performance on the reading measures. Correlational studies in adults have shown that musicians have greater sensitivity to speech rhythm (Marie et al., 2011), better reading-related skills (e.g., phoneme discrimination: Zuk et al., 2013a) and that individual differences in speech rhythm sensitivity is related to variability in musical aptitude when participants with a wide range across the continuum of musical abilities are studied (Magne et al., in revision). Over the course of aging, there is evidence that early musical training is associated with protection against age-related linguistic and cognitive declines (Parbery-Clark et al., 2011; Bidelman et al., 2014; Bidelman and Alain, 2015), even in adults with hearing loss (Parbery-Clark et al., 2013). However, as noted in Butera (2015), associations with musical training in these correlational studies cannot be interpreted in favor of causality in the absence of longitudinal data that rules out other genetic and environmental contributions to the observed findings of neural enhancements in individuals with musical training.

If enhanced language skills and musical skills are correlated, then would individuals with language disorders also have deficits in musical processing? Research on reading disabilities and language impairment suggests that this is often the case (e.g., Goswami, 2011; Gordon et al., 2015a). Seminal work by Overy (2003) revealed that a small group of children with reading disability improved their phonological awareness and spelling skills faster during an 8-week period of music instruction than during the same amount of time with no music training. Sensitivity to musical rhythm predicted significant variance in phonological awareness concurrently and longitudinally in 10-year-olds with dyslexia (Huss et al., 2011; Goswami et al., 2013). Difficulties processing the prosodic aspect of speech (i.e., variations in timing and pitch that mark linguistic events) are thought to be reflected in both musical deficits and weaknesses in phonological awareness (Goswami et al., 2010; Power et al., 2013; Leong and Goswami, 2014) in individuals with reading disabilities. Given these connections, musical practice holds promise as a tool to contribute to reading skills, potentially via a pathway of enhancing children's sensitivity to prosodic aspects of speech.

Correlational evidence does not, of course, exclude potential effects of self-selection or environmental and genetic differences that could alternatively account for enhanced language skills in musicians (Schellenberg, 2015). Evidence from longitudinal studies that administer a controlled and specific amount of musical training is crucial for investigating a possible causal influence of music on non-musical skills. The potential that music training could enhance reading skills is especially pertinent now that there are ongoing debates in educational systems about the most effective strategies for impacting academic achievement in the core curriculum. However, it is important to note that much of this work has focused on training-related brain changes (rather than behavioral outcomes); the significance for academic achievement of these modifications in brain activity is difficult to ascertain in the absence of reporting of behavioral gains in language skills (as discussed in Evans et al., 2014; Schellenberg, 2015). As reviewed in the present study, a considerable collection of controlled training studies has provided positive evidence for the hypothesis that musical training transfers to literacy-related skills. Taken as a whole, however, the range of studies published to date present a rather mixed set of results, marked by a large range of potential outcome measures related to literacy skills. To assess and quantify the state of the evidence that may potentially support the hypothesis that musical training in children transfers into enhancements in literacy-related skills, we first set out to delineate the subset of peer-reviewed papers that directly address this issue via training and pre- post-assessment designs.

A meta-analytic approach is useful in assessing the efficacy of music training for language outcomes and identifying the attributes of music training paradigms that are relevant to specific reading outcomes. The present meta-analysis is thus aimed at synthesizing previous research on music training and reading-related outcomes. The following research questions were examined:

1. Does music training improve reading-related outcomes when other reading instruction is controlled for? Are certain aspects of learning how to read (i.e., reading fluency and phonological awareness) particularly susceptible to transfer from music training?
2. Does the age of participants account for variability in the efficacy of the training?
3. Does the quantity of music training impact the efficacy of the training, and how many hours of training are needed to affect changes in reading-related outcomes?
4. Does the design of the control group condition moderate outcomes?

## Materials and methods

### Literature search

#### Search strategies

The goal of this meta-analysis is to evaluate the effectiveness of musical interventions on reading-related measures. To find all articles that met our criteria, we conducted a literature search using the PubMed, Web of Knowledge, and ProQuest article databases. ProQuest functioned as a meta-database, allowing us to search 12 databases simultaneously: ERIC, International Index to Music Periodicals Full Text, Linguistics and Language Behavior Abstracts, MLA International Bibliography, ProQuest Education Journals, ProQuest Psychology Journals, ProQuest Research Library, ProQuest Science Journals, ProQuest Social Science Journals, PsychARTICLES, PsycINFO, and RILM Abstracts of Music Literature. The search terms used in each of the three searches are listed in Supplementary Table 1. The initial search was conducted in November 2013, and it was repeated/updated in March 2014. In total, the search returned 4855 articles whose article titles were searched for relevance to the topic. Additionally, to pass this first screening phase, each article could not be a conference presentation, thesis or dissertation, or trade newspaper or magazine article, and had to be written in English. A preliminary search of these titles narrowed down the potentially relevant articles to 178. The abstracts of these remaining articles were then reviewed for inclusion criteria and relevance. The criteria in this second phase of screening required that articles not be a review or meta-analysis, that they have a music intervention with a control group, and that they investigated reading-related outcomes.

#### Inclusion and exclusion criteria

In our literature review, we defined inclusion and exclusion criteria based on meta-analysis guidelines for distinguishing features of studies (e.g., characteristics of the participants, key variables, research methods, and publication type; Lipsey and Wilson, 2001). Only articles that met the following criteria were included in the study:

1. *Had an intervention with a control group* (i.e., no within-group interventions, observational or correlational studies).
2. *Was a peer-reviewed publication* (i.e., no dissertations/thesis, conference proceedings, unpublished manuscripts, or secondary sources such as trade magazines or media coverage). This criteria was adapted to ensure a minimally acceptable level of quality and rigor. This approach coincides with the National Reading Panel's standards for meta-analysis (Lonigan and Shanahan, 2009) and with previous meta-analysis on literacy education (e.g., Bus and van IJzendoorn, 1999).
3. *Reported phonological or reading-related outcomes*.
4. *Assessed outcomes pre- and post-intervention*.
5. *Provided sufficient data to extract effect sizes (means, SD, and N, pre- and post-intervention, for the same participants in each group)*. For studies that met requirements 1 through 4 but did not report sufficient data in the published paper, corresponding authors were contacted via email and asked to provide the additional information. In two cases (Douglas and Willatts, 1994; Standley and Hughes, 1997), authors responded that the data was not available due to the long time period that has lapsed since the publication of their respective studies; thus these two studies were excluded.

Out of 178 studies that were reviewed at the abstract level (with full-text examination if necessary to determine inclusion based on above criteria), 17 articles met these criteria. The types of interventions used and contrasting control groups were found to vary substantially across the studies, with some showing confounds of uneven amounts of reading instruction across the groups or failed to provide more musical training to one of the groups. We thus added the following constraint to study design for inclusion:

1. The intervention group had to receive more music instruction than the control group.
2. Studies need to provide an indication of equivalent amounts of reading instruction across the intervention and control groups.

After applying this final design constraint, an additional 5 studies were excluded (Register, 2001; Register et al., 2007; Bolduc, 2009; Darrow, 2009; Bhide et al., 2013) and only 12 papers still qualified, as listed in Table 1 (Register, 2004; Gromko, 2005; Myant et al., 2008; Moreno et al., 2009, 2011; Yazejian and Peisner-Feinberg, 2009; Degé and Schwarzer, 2011; Herrera et al., 2011; Bolduc and Lefebvre, 2012; Cogo-Moreira et al., 2013; Moritz et al., 2013; Thomson et al., 2013). Herrera et al. presented results from two independent samples (each with its own control group) that received the same intervention, and was thus coded as two separate studies in our analysis, giving a final study count of *k* = 13 for the meta-analysis.

**Table 1:** **Study characteristics**.

**Study, Year, Journal**	**Language**	**Mean age of participants**	**Reading fluency outcome measure**	**Phonological outcome measures**	
				**Rhyming**	**Other phonological measures**
(Bolduc and Lefebvre, 2012), Creative Education	French	4.9 (*N* = 54)			Phonological awareness measure (PAM; Armand and Montésinos-Gelet, 2001)
(Cogo-Moreira et al., 2013), PLoS ONE	Portuguese (Brazil)	9.2 (*N* = 235)	Accuracy of Word reading (custom)		Test of phonological awareness (Capovilla and Capovilla, 1998)
(Degé and Schwarzer, 2011), Frontiers in Psychology	German	5.8 (*N* = 27)			Phonological awareness—total from Bielefelder screening (Jansen et al., 2002)
(Gromko, 2005), Journal of Research in Music Education	English (US)	5.5 (*N* = 103)	DIBELS letter-naming fluency (Good and Kaminski, 2002)		DIBELS phoneme-segmentation fluency
(Herrera et al., 2011), Psychology of Music	Spanish	4.5 (*N* = 29)		Rhyme oddity task (custom)	Initial phoneme oddity task (custom)
(Herrera et al., 2011), Psychology of Music	Tamazight	4.7 (*N* = 27)		Rhyme oddity task (custom)	Initial phoneme oddity task (custom)
(Moreno et al., 2009), Cerebral Cortex	Portuguese (Portugal)	8.3 (*N* = 32)	Reading inconsistent words (from Portuguese European reading battery, Succena and Castro, 2010)		
(Moreno et al., 2011), Music Perception	English (Canada)	5.3 (*N* = 60)		Rhyming (from WJ-III, Woodcock et al., 2001)	
(Moritz et al., 2013), Reading and Writing	English (US)	5.6 (*N* = 30)		Rhyming Discrimination from Phonological awareness test (PAT; Robertson and Salter, 1997)	Isolation of initial phonemes from PAT
(Myant et al., 2008), Educational and Cognitive Psychology	English (UK)	4.3 (*N* = 59)		Rhyme test from Phonological Assessment Battery (PhAB; Frederickson et al., 1997)	Alliteration test from PhAB
(Register, 2004), Journal of Music Therapy	English (US)	5.5 (*N* = 43)	Letter-naming fluency from DIBELS (Good and Kaminski, 2001)		Initial sounds fluency from DIBELS
(Thomson et al., 2013), Reading and Writing	English (UK)	9.3 (*N* = 21)	TOWRE (Torgesen et al., 1999)	Rhyme test from PhAB	Spoonerisms from PhAB
(Yazejian and Peisner-Feinberg, 2009), NHSA Dialog	English (US)	4.4 (*N* = 181)		Rhyming from Early Phonological Awareness Profile (EPAP; Dickinson and Chaney, 1997)	Phoneme deletion from EPAP

*Study information, primary language of participants, age, and outcome measures of studies included in the meta-analyses*.

### Coding procedures

#### Procedure and outcome variables

A custom data entry system was created for the study using the Research Electronic Data Capture (REDCap) tools (Harris et al., 2009) hosted at Vanderbilt University (REDCap is a secure, web-based application designed to support data capture for research studies, providing an intuitive interface for validated data entry and automated export procedures for seamless data downloads to common statistical packages). All study characteristics and data were coded and entered into the custom forms.

The outcomes measures used within these 13 studies are somewhat variable; each can be classified into one of the two broad categories of Reading Fluency and Phonological Awareness. For studies that reported more than one measure in an outcome category, we selected the measure that most directly tapped into the category. For Reading Fluency, measures that emphasized fluent use of known words and letters were chosen over those that used non-words. Within Phonological awareness, two subcategories were identified: Rhyming and Other Phonological measures. For Rhyming, measures that involved discrimination of rhymes were chosen over those that involved producing rhymes. For Other Phonological, measures that involved identification, discrimination, or manipulation of phonemes were chosen over those that dealt with non-word reading fluency or syllabic segmentation. All measures included are reported in Table 1.

#### Potential moderating variables

These 13 studies were then carefully coded for the following study design features, which are reported in Tables 2, 3.

1. *Total number of hours in music intervention*.
2. *Type of Control Intervention*. Control interventions included: phonological control, non-auditory control (sports or art), less intensive music control, and no-treatment control. In studies that included more than one intervention or control group, only the group that fully met the requirements outlined in the Inclusion and Exclusion Criteria section was included.
3. *Level of Random Assignment employed*. The following types of assignment were indicated: Student Random (children were enrolled in the study and assigned randomly to intervention and control groups); Random or Non-random Assignment by Class (multiple classes within a school were enrolled in the study and full classes were assigned to the intervention randomly or non-randomly); Random or Non-random Assignment by School (multiple schools were enrolled in the study and participants were assigned to the intervention randomly or non-randomly).
4. *Components of Music Training*. The following component categories of musical activities were coded in a binary manner (i.e., we coded whether or not the intervention included each component): Phonology in Musical context; Gross motor Movement/Kinesthetic activities; Rhythm; Musical Instruments; Rhyming; Clapping/Marching; Visual representations of musical concepts (i.e., visual portrayals of high vs. low pitch or short vs. long sounds); Singing; and Musical notation.
5. *Mean age of participants*.
6. *Subject population (Typically or Atypically developing)*.
7. Was Socio-economic status (SES) reported and controlled for across groups?
8. Was IQ controlled reported and controlled for across groups?

**Table 2:** **Training components**.

**Study**	**Total hours of training**	**Training components**								
		**Phonology in music context**	**Movement/Kinesthetic**	**Rhythm**	**Instruments**	**Rhyming**	**Clapping/Marching**	**Visual representations**	**Singing**	**Musical notation**
Bolduc and Lefebvre, 2012	6.67	✓			✓	✓			✓	
Cogo-Moreira et al., 2013	50		✓	✓	✓				✓	
Degé and Schwarzer, 2011	16.67		✓	✓	✓				✓	✓
Gromko, 2005	6.5		✓	✓	✓			✓	✓	
(Herrera et al., 2011), Spanish	16	✓				✓	✓	✓	✓	
(Herrera et al., 2011), Tamazight	16	✓				✓	✓	✓	✓	
Moreno et al., 2009	60	✓	✓	✓	✓	✓	✓		✓	✓
Moreno et al., 2011	40			✓					✓	✓
Moritz et al., 2013	90		✓	✓					✓	
Myant et al., 2008	17.5		✓	✓			✓		✓	
Register, 2004	8.5	✓	✓		✓				✓	
Thomson et al., 2013	3	✓		✓			✓	✓		
Yazejian and Peisner-Feinberg, 2009	26		✓	✓	✓	✓		✓	✓	

*Hours of music training and components of the music intervention for each study*.

**Table 3:** **Study controls**.

**Study**	**Subject population**	**Control for IQ**	**Control for SES**	**Type of assignment**	**Control interventions**
Bolduc and Lefebvre, 2012	Typical	Yes	SES not reported	Random assignment by class	Phonological control
Cogo-Moreira et al., 2013	Atypical	Yes	SES not reported	Random assignment by school	No-treatment control
Degé and Schwarzer, 2011	Typical	Yes	Yes	Student random	Non-auditory control (sports)
Gromko, 2005	Typical	IQ not reported	No	Non-random assignment by school	No-treatment control
Herrera et al., 2011, Spanish	Typical	Yes	SES not reported	Student random	Phonological control
Herrera et al., 2011, Tamazight	Typical	Yes	SES not reported	Student random	Phonological control
Moreno et al., 2009	Typical	Yes	Yes	Student random	Non-auditory control (art)
Moreno et al., 2011	Typical	Yes	Yes	Student random	Non-auditory control (art)
Moritz et al., 2013	Typical	Yes	No	Non-random assignment by school	Less intensive music control
Myant et al., 2008	Typical	IQ not reported	Yes	Non-random assignment by school	No-treatment control
Register, 2004	Typical	IQ not reported	Yes	Non-random assignment by class	No-treatment control
Thomson et al., 2013	Atypical	Yes	SES not reported	Student random	No-treatment control
Yazejian and Peisner-Feinberg, 2009	Typical	IQ not reported	Yes	Random assignment by class	No-treatment control

*This table reports population, IQ, SES, type of assignment, and control interventions for each study*.

### Statistical analysis

#### Effect size calculation

For each outcome and measure, a single effect size was computed in the following manner, where ES = effect size:

$$\text{ES}=\frac{\left(\text{Posttest MeanTx }-\text{ Pretest MeanTx})\;-\;(\text{Posttest MeanControl }-\text{ Pretest MeanControl}\right)}{\text{Pooled Pretest SD}}$$

$$\text{Pooled Pretest SD}=\sqrt{\frac{\text{Pretest }{\text{SD}}_{Tx}{}^{2}\;*\;(N_{Tx}\;-\;1)\;+\text{Pretest }{\text{SD}}_{Control}{}^{2}*\left(N_{Control}\;-\text{ 1}\right)}{N_{Tx}\;+\;N_{Control}-2}}$$

#### Data-analysis

Meta-analysis was performed using the open-source statistical software package R (R Core Team, 2015), and employing the “*metafor*” package (Viechtbauer, 2010). Heterogeneity was computed as *I*^2^ = residual heterogeneity divided by unaccounted variability, and *H*^2^ = unaccounted variability divided by sampling variability (Higgins and Thompson, 2002). Meta-analysis was carried out using two different approaches: random effects model for the separate analysis of each of the three outcome types (Reading Fluency, Rhyming, and Other Phonological outcomes), and mixed effects model for the moderator analysis. Mixed effects was also used for the broader All Phonological Outcomes category since it included non-independent samples from studies that included both Rhyming and Other Phonological Outcomes. Moderator analysis was used to test influence of age, control intervention type, and number of training hours on the efficacy of music interventions. Given the relatively small number of studies included in the meta-analysis, it was not possible to test additional moderators for each component of training and level of random assignment.

## Results

### Characteristics of the studies included

Publication information, language, age of participants, and outcomes measured are reported in Table 1. Participants ranged in mean age from 4.53 to 9.33 years, with a weighted average mean of 6.25. Participants identified with a wide range of native languages (English, Portuguese, German, French, Spanish, and Tamazight). The components of music training are reported in Table 2 and varied greatly across studies; total hours of training ranged from 3 (Thomson et al., 2013) to 90 (Moritz et al., 2013). Many studies included singing (*k* = 12), rhythm (*k* = 9), instruments (*k* = 7), movement/kinesthetics (*k* = 8), and less than half used Phonology in music context (*k* = 6), rhyming (*k* = 5), clapping/marching (*k* = 5), visual representations of musical concepts (*k* = 5), and only *k* = 3 included music notation.

Several aspects of control factors in the study design are reported in Table 3. All but two studies (Cogo-Moreira et al., 2013; Thomson et al., 2013) were conducted on a typically developing children. IQ was reported as equivalent across groups in *k* = 9 studies, and SES was reported as equivalent across groups in only *k* = 6 studies. Many different types of group assignment were found, and only *k* = 6 studies used “true” student random assignment. The remaining studies assigned pre-existing classes (or schools) to different treatment conditions. Control interventions included *k* = 3 studies in which the control group received phonological training, *k* = 3 studies with non-auditory control activities such as art or sports, *k* = 6 studies with no special extra-curricular activities (no-treatment control), and one study where the control group also received music lessons but to a much lesser extent (“less intensive music” control).

### Effect sizes

Means, standard deviations, pre- and post-training, N's per group, and the computed effect sizes are reported in Table 4. Given that this meta-analysis was designed to investigate (1) how music training affects different types of reading-related measures; and (2) how selected aspects of study design (age of participants, hours of training, and type of control intervention) would moderate outcomes, the choice to limit the moderator analysis to these three moderator variables was also constrained by the statistical power of conducting meta-regression on only a small number of studies that met the criteria. Thus, meta-analyses were computed separately on reading fluency and phonological awareness, and moderator analyses tested the influence of each of the abovementioned factors on the outcomes.

**Table 4:** **Effect sizes**.

**Study**	**Measure**	***N* music**	**Music group pre-training Mean (*SD*)**	**Music group post-training Mean (*SD*)**	***N* control**	**Control group pre-training Mean (*SD*)**	**Control group post-training Mean (*SD*)**	**Standardized effect size**
**READING FLUENCY DATA**								
Cogo-Moreira et al., 2013	Accuracy of word reading	114	9.45 (11.33)	16.14 (15.59)	121	11.22 (14.37)	16.5 (15.33)	0.11
Gromko, 2005	DIBELS letter-naming task	43	33.42 (15.48)	42.63 (15.22)	60	36.27 (18.87)	44.1 (15.63)	0.08
Moreno et al., 2009	Inconsistent word reading	16	41.73 (16.38)	71.35 (13.25)	16	45.83 (17.48)	56.77 (17.53)	1.07
Register, 2004	DIBELS letter naming fluency	22	12.18 (10.58)	20.23 (14.27)	21	17.48 (16.74)	25.38 (17.65)	0.01
Herrera et al., 2011	Word reading	9	49.67 (12.44)	52.44 (11.26)	12	48 (15.97)	48.25 (17.27)	0.17
**OTHER PHONOLOGICAL DATA**								
Bolduc and Lefebvre, 2012	Phonological Awareness Measure (PAM)	28	10.5 (2.58)	14.8 (3.65)	26	12 (3.12)	15.4 (3.54)	0.31
Cogo-Moreira et al., 2013	Phonological awareness	114	25.79 (4.96)	27.66 (4.64)	121	23.98 (5.13)	25.18 (5.25)	0.13
Degé and Schwarzer, 2011	Phonological Awareness—Total	13	35.77 (2.35)	38.23 (1.17)	14	35.86 (3.18)	36.07 (2.99)	0.78
Gromko, 2005	DIBELS phoneme-segmentation fluency	43	18.61 (16.26)	44.72 (16.94)	60	25.83 (14.73)	41.55 (14.5)	0.67
(Herrera et al., 2011), Spanish	Initial sound	15	42.69 (22.5)	60.14 (12.5)	14	45.8 (17.76)	60.5 (12.63)	0.13
(Herrera et al., 2011), Tamazight	Initial sound	17	42.44 (10.2)	51.99 (8.67)	10	39.72 (12.18)	55.14 (7.9)	–0.52
Moritz et al., 2013	PAT isolation initial	15	7.5 (2.15)	9.93 (0.27)	15	6.57 (2.3)	9.15 (1.21)	–0.07
Myant et al., 2008	Alliteration	28	1.82 (2.58)	3.35 (3.35)	31	0.26 (0.58)	1.11 (1.6)	0.37
Register, 2004	DIBELS initial sounds fluency	22	6 (6.62)	14.27 (8.47)	21	9.52 (6.41)	15.71 (8.04)	0.31
Thomson et al., 2013,	PhAB spoonerisms	9	14.11 (6.54)	17.44 (7.38)	12	14.17 (7.21)	14.83 (6.93)	0.37
Yazejian and Peisner-Feinberg, 2009	Phoneme deletion	111	10.35 (4.19)	12.32 (2.88)	70	8.99 (4.68)	12.03 (3.27)	–0.24
**RHYMING DATA**								
(Herrera et al., 2011), Spanish	Rhyme oddity	15	42.08 (11.97)	56.64 (6.82)	14	40.56 (14.49)	52.49 (10.94)	0.19
(Herrera et al., 2011), Tamazight	Rhyme oddity	17	46.68 (8.8)	64.65 (9.12)	10	42.92 (11.4)	57.36 (10.27)	0.35
Moreno et al., 2011	Rhyming	30	9.2 (2.9)	11 (3.7)	30	8.6 (3.9)	10 (4.3)	0.11
Moritz et al., 2013	PAT rhyming discrimination	15	7.53 (2.1)	9.86 (0.36)	15	8.64 (1.39)	8.77 (1.54)	1.20
Myant et al., 2008	Rhyme	28	3.86 (2.92)	6.77 (3)	31	3 (2.67)	6.04 (2.93)	–0.05
Thomson et al., 2013	PhAB rhyme	9	16.78 (2.28)	18.78 (2.28)	12	14.08 (5.45)	15.08 (5.87)	0.22
Yazejian and Peisner-Feinberg, 2009	Rhyme recognition	111	3.52 (2.89)	6.05 (3.74)	70	2.76 (2.58)	5.21 (3.85)	0.02

*Means and SD's for each group, and effect sizes, are listed for each study (grouped by outcome category type)*.

### Meta-analysis results for phonological awareness

**Figure 1:**
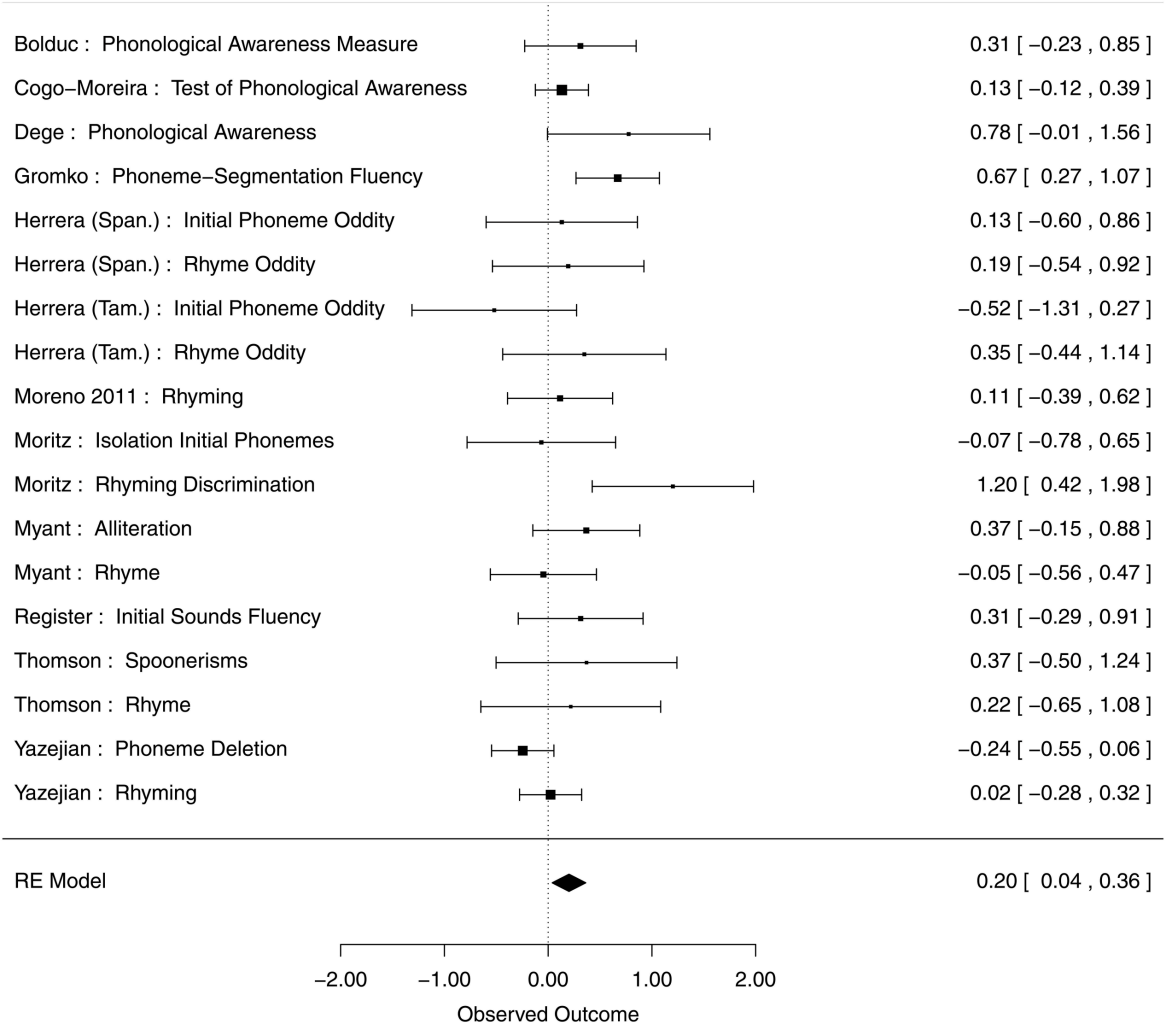
**Influence of music training on Phonological Awareness outcomes**. The forest plot shows weighted effect sizes for music vs. control group on all phonological outcomes, in each study, and across studies. Confidence intervals are given in brackets.

Due to the non-independence of the studies that reported both types of phonological awareness outcomes (Rhyming and Other Phonological) in the same sample, mixed effects analysis was employed to test overall Phonological Awareness. This analysis on All Phonological Awareness (*k* = 18) revealed an effect size of 0.20 (95% CI [0.04, 0.36], *p* = 0.01), showing small but significant gains of music training on phonological skills, shown in the forest plot in Figure 1. The test for Heterogeneity [*Q*_(*df* = 17)_ = 28.8, *p* = 0.04] was significant, indicating potential influence of other factors. To investigate these factors and their relation with moderators, phonological outcomes were then further broken down into two separate categories corresponding to Rhyming and Other Phonological outcomes (see Methods section for more information on how measures/outcomes were chosen).

#### Rhyming outcomes

**Figure 2:**
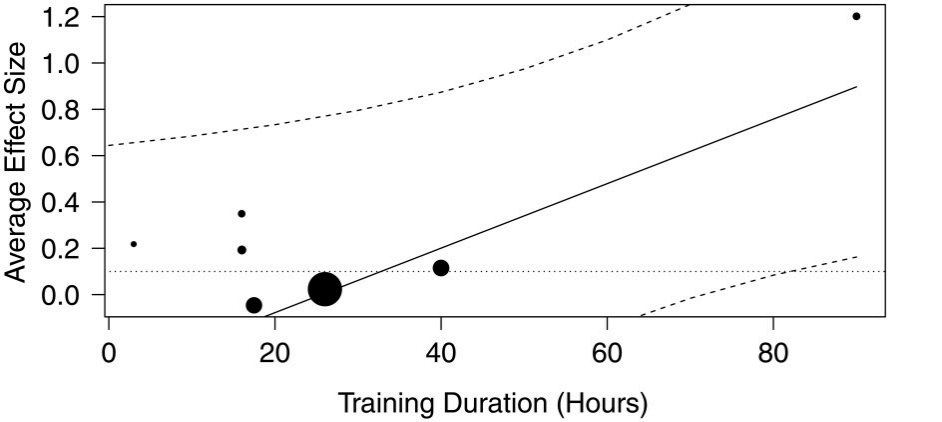
**Music training duration moderates intervention efficacy**. The plot shows the average effect sizes (y-axis) vs. training duration (moderator variable), based on a model estimating that a minimum of 40 h of music training is needed to improve rhyming skills.

Random-effects analysis on the subset of rhyming outcomes (*k* = 7 studies) yielded a weighted average effect size of 0.18 (95% CI [−0.06, 0.42]), which was non-significant at *p* = 0.14. A mixed effects analysis then revealed no significant influence of age (*p* = 0.31) or control intervention type (*p* = 0.75) on the results, but a significant influence (*p* = 0.04) of training hours on rhyming outcomes. These results suggest that an increase in the length of training by 1 h corresponds to an increase of 0.01 (95% CI [0, 0.03]) in the effectiveness of music intervention on rhyming outcomes. The results of this model were then used to predict values of effectiveness given different amounts of training hours. Using the range of values from across all studies from the entire meta-analysis, (3–90 h), and assuming a constant age (5 years) and constant control intervention type, the model predicts that at least 40 h of training are needed to have a significant effect on Rhyming outcomes, as shown in Figure 2. These results should be interpreted with caution, given that the study showing the strongest positive relationship between hours of training and rhyming outcomes (Moritz et al., 2013) had only 15 participants in each group.

#### Other phonological outcomes

Random effects analyses on Other Phonological outcomes (*k* = 11), yielded an average effect size of 0.20 (95% CI [−0.03, 0.42]), which weakly trended toward significance (*p* = 0.08). A mixed effects analysis revealed no significant influence of age (*p* = 0.24), control group type (*p* = 0.34), or training hours (*p* = 0.09) on the model. Heterogeneity was moderate (*I*^2^ = 40.2%; *H*^2^ = 1.67) but residual heterogeneity did not reach significance [*QE*_(*df* = 7)_ = 11.89, *p* = 0.10].

### Meta-analysis results for reading fluency

**Figure 3:**
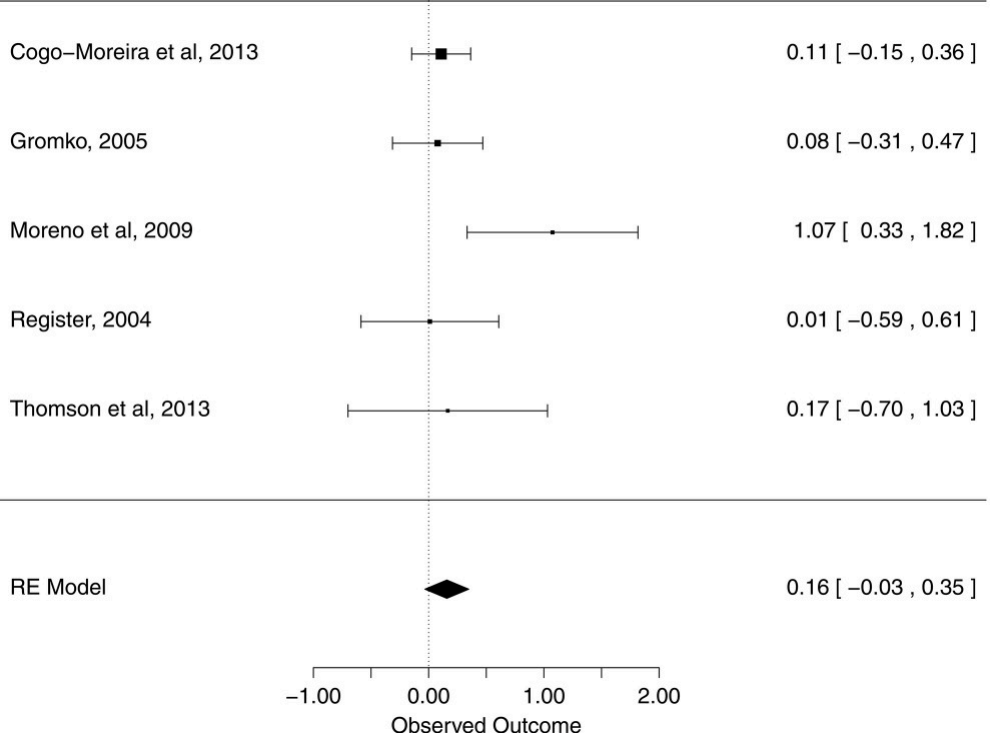
**Influence of music training on Reading Fluency outcomes**. The forest plot shows weighted effect sizes for music vs. control group on reading fluency outcomes, in each study, and across studies. Confidence intervals are given in brackets.

Random effects analysis on the five studies that included Reading Fluency outcomes showed a weighted average effect size of 0.16 (95% CI [−0.03, 0.35], *p* = 0.10), thus showing only a weak trend toward significance of music intervention on reading fluency. Results are shown in Figure 3. Heterogeneity was low (*I*^2^ = 0%; *H*^2^ = 1), and given the small number of studies (*k* = 5), moderator analysis was not pursued.

### Test for publication bias

The Rank Correlation Test for Funnel Plot Asymmetry indicated no publication bias for either Reading Fluency (Kendall's tau = 0.60, *p* = 0.23) or Phonological Awareness (Kendall's tau = 0.18, *p* = 0.33).

## Discussion

The current meta-analysis was carried out to assess the impact of music intervention on reading-related skills in children, and adds to the literature by specifically highlighting effects of music training transferring to reading-related skills when non-musical reading training is held constant. Results of the meta-analysis on the broad category of Phonological Awareness outcomes suggest modest gains (a small effect size of *d* = 0.20) for music vs. control groups. This finding is in line with a number of other studies showing better phonological awareness skills in musicians compared to their non-musician peers (Forgeard et al., 2008; Zuk et al., 2013b), and also converges with work showing correlations between music aptitude and phonological skills in children (Lamb and Gregory, 1993; Anvari et al., 2002; Peynircioglu et al., 2002; Dellatolas et al., 2009; Tierney and Kraus, 2013a).

When broken down into subcategories (Rhyming and Other Phonological outcomes), moderator analysis revealed that the effectiveness of music intervention on Rhyming outcomes was dependent on the number of training hours. Total music intervention training hours ranged between 3 and 90 h in the studies included here, and the model estimated that at least 40 h are needed to improve Rhyming skills. To put this number in perspective, other work (e.g., Hambrick et al., 2014) has shown that thousands of hours are typically involved in reaching adult levels of musical expertise. Consideration of how children's music training improves rhyming skills must assess the possibility that results could merely reflect the inclusion of greater rhyming practice within the music interventions relative to the control conditions. Indeed, early childhood music education in group settings typically include activities such as singing and chanting rhyming lyrics. However, several aspects of the studies that support the positive transfer effect for rhyming outcomes suggest that this effect cannot be entirely attributed to this explanation. First, it should be noted that the study with the strongest positive relationship between rhyming outcomes and hours of training (Moritz et al., 2013) reported no rhyming-related training activities, and rather emphasized rhythmic aspects of musical training. Furthermore, of the seven studies with rhyming outcomes, only four were coded as including any report of rhyming training (see Table 2). These results, taken together with reports of robust associations between musical rhythm skills and rhyme awareness, in both children with typical development and reading disabilities (e.g., Huss et al., 2011), suggest that other aspects of musical training may impact rhyming skills. Future work is needed to make more definitive conclusions regarding whether intensive rhythm training can improve rhyming and phonological skills in general, given the links between rhythm and reading skills in the literature (e.g., Strait et al., 2011).

The separate meta-analysis on eleven datasets with Other Phonological Outcomes was inconclusive: the effect size was small (*d* = 0.2) and only trended toward significance, with no moderators (age, control intervention type, or training hours) reaching significance. This pattern of results could potentially be due to variability in the many different types of phonological tasks that were included in this category (i.e., Initial Phoneme Oddity, Alliteration, Spoonerisms and others; see Table 1) or even to the wide variety of native languages spoken by participants. Further study is needed to determine if certain phonological skills are more susceptible to a positive transfer from music training than others.

The effect size for the separate meta-analysis assessing the impact of music training on reading fluency outcomes was also small (*d* = 0.16) and did not reach significance: moderator analysis was precluded due to only having five studies in this category. However, it should be pointed out that two of the studies (Cogo-Moreira et al., 2013; Thomson et al., 2013) were on children with reading disabilities, and while there are solid theoretical reasons (see Overy, 2003; Tierney and Kraus, 2013a) to believe that music training could improve reading skills in struggling readers, the intensity of the intervention would likely be an important factor in such attempts.

Moreover, previous meta-analyses with different parameters than the present study have found both a non-significant effect of music on reading skills (Butzlaff, 2000) and significant effects (Standley, 2008). The present study extends these results by including data from additional studies published between 2008 and 2014, and by limiting the scope of studies included to a more rigorously defined comparison, for which reading instruction is controlled across groups. The data quality and variability of study outcomes and confidence intervals are comparable to studies included in other meta-analyses on literacy education (e.g., Lonigan and Shanahan, 2009) and this heterogeneity should be taken into account in the interpretation (as discussed below).

It is interesting to note that a previous meta-analysis on literacy development found medium-to-strong effects of phonological awareness training on reading skills (yet longer term studies produced only small effects), and that phonological awareness was a necessary, but not sufficient condition for reading (Bus and van IJzendoorn, 1999). One could hypothesize that music skills share more variance with phonological skills (due to their auditory bases) than with reading fluency skills, and thus music training may have larger effects on phonological awareness than on reading. Nonetheless, it is also possible that music training could impact reading fluency via a more gradual pathway: beginning more generally by improving auditory discrimination, then affecting rhyming skills and using them to bootstrap further phonological awareness. More intensive training may be needed for these improvements to occur at a level that produces measurable improvements in reading fluency across heterogeneous participant populations.

Overall, the findings of the current meta-analyses are somewhat inconclusive with regards to the hypothesized impact of music education on reading-related skills. The literature search revealed a large amount of variability in outcomes studied, content and intensity of music training, native language of participants, type of subject populations (typically developing vs. reading disordered) and age of participants. In addition, some of the study designs in the set of studies included in this meta-analysis are laden with potential biases that make it difficult to draw broader conclusions from the findings (see Table 3). These inconsistencies include variability in control group activities, lack of information about IQ differences or equivalence across groups; and only 6 studies of 12 reported controlling for socio-economic status across groups. Importantly, most of the studies were quasi-experimental and did not use random assignment to create treatment and control groups. In the case of studies that compared a class (or school) receiving the intervention vs. another control class or school, it is possible that other differences in teacher/student dynamics and educational environment differed across the groups (and therefore either diminished or exaggerated the gains in music training). Although we were able to code and report many of the above characteristics, there were too few studies included in the total meta-analysis to allow a sufficiently powered moderator analysis that would effectively shed light on whether these study characteristics were linked with different trends of results. Thus, the limitations of the present meta-analysis are the heterogeneity of approaches and study designs used, and that the dataset was too underpowered to test all of the potentially influential moderator variables that were coded. Nevertheless, it is interesting to note that all three of the studies (Moreno et al., 2009, 2011; Degé and Schwarzer, 2011) in which SES and IQ were equivalent, and student random assignment was used, also showed large effect sizes on at least one reading-related outcome, indicating a robustness of music training efficacy for improving reading-related skills under methodologically sound circumstances. The quality and breadth of all studies included in the present meta-analyses also provides complementary information to results of a prior meta-analysis on the impact of music on reading skills (i.e., Standley, 2008) in which aspects of the music training may have confounded the findings (e.g., some studies included in their meta-analysis included contrasts where both groups received different types of music training and whether a given group got more music training was unclear). Suggestions for creating a standard of implementation steps for reducing heterogeneity and bias are summarized in Table 5.

**Table 5:** **Future directions for studying the impact of music education on reading skills**.

**Factors to control**	**Questions of interest to test in future studies**
• IQ and socio-economic status	• What are the effects of different components of interventions (rhythm, pitch; instruments vs. singing; phonological activities in musical context, etc.) on training efficacy?
• Control intervention content	• What degree of music-driven gains in phonological awareness are needed to impact reading fluency?
• Type and duration of music training	• What are the mechanisms underlying improvement: such as attention, motivation, (e.g., OPERA hypothesis; Patel, 2011), speech prosody sensitivity, and/or working memory?
• Guidelines for typical and atypical development	• How are changes in brain function and structure associated with music-training-driven improvements?
• Random assignment to experimental groups	• How do individual differences predict response to training? Is there a subset of children that stands to benefit the most from music training?

Moreover, the small effects of music on reading-related outcomes observed in this meta-analysis stand in contrast to the robust results seen in the correlational literature reporting (broadly defined) linguistic advantages in musician children (Magne et al., 2006; Chobert et al., 2011) and adults who had musical training as children (Skoe and Kraus, 2012). One key difference is that the correlational studies tend to include children who have already had several years of individual instrumental instruction, whereas the intervention studies included here have shorter and less intense music training, and all were conducted in a group rather than individual setting. It could be that the music training in a group setting is less demanding and therefore less likely to make a large impact in terms of transferring to language skills (see OPERA hypothesis for a theoretically driven set of criteria for plasticity; Patel, 2011). Nevertheless, Hyde et al. (2009) showed neural plasticity and improvements in auditory and motor tasks, along with structural brain changes in auditory and motor areas, after 15 months of music training on an instrument. Furthermore, other experimental studies administering group-setting music training to participants randomly assigned to a music group (vs. a non-music control group, i.e., Moreno et al., 2009; Chobert et al., 2014) also found transfer to language perception skills; thus, individual instruction does not appear to be a pre-requisite for music-training-driven improvements in language skills. However, less is known about whether individual lessons and intensive instruction on an instrument are needed to improve reading-related skills.

The literature review encompassed by the present study revealed two somewhat opposing trends: on the one hand, an approach that favors *the contextual use of music* as a fun and motivational context to teach reading and other skills (Standley and Hughes, 1997; Standley, 2008; Darrow, 2009); and on the other hand, an *auditory neuro-development framework* that attributes music-training-related language gains primarily to auditory neural plasticity (Kraus and Chandrasekaran, 2010; Patel, 2011). In the “contextual” approach, phonological awareness and other literacy skills are taught in a musical context: for example, one intervention was described as teaching “literacy skills such as rhyming, letter sounds, vocabulary, or decoding sounds that were accompanied by a chant or song; children's storybooks that were either read or sung or accompanied by the students on musical instruments as they recognized a previously identified vocabulary word; rearrangement of storybook parts with students asked to put the story pages in order and to retell the story in their own words” (Darrow, 2009, p. 14). Use of nursery rhymes is common and constitutes the foundation of one of the intervention curricula described in a study in the meta-analysis (Bolduc and Lefebvre, 2012). A number of studies have specifically targeted literacy skills within the music training, with musical activities designed to increase print awareness (Standley and Hughes, 1997); letter-naming, letter-sound correspondence, and word building (Register, 2004); and decoding (Register et al., 2007). Interestingly, in many of the *contextual* studies, music is thought of as a positive reinforcer of reading-related exercises, and little mention is made of the auditory system or its physiological underpinnings.

In contrast, the auditory neurodevelopment framework posits that music training strengthens basic auditory and speech processing, which in turn influence phonological perception and reading skills. These gains have been described as domain-general improvements in auditory brain mechanisms underlying temporal and frequency resolution, auditory processing, and phonological awareness (Tierney and Kraus, 2013a). Experience-based plasticity of brain networks involved in language acquisition is a plausible explanation for the putative transfer of music training to language and literacy skills (reviewed in Kraus and Chandrasekaran, 2010). Randomized study designs conducted with neuro-imaging methods have shown that music lessons (in typically developing children) enhance neural responses to voice-onset-times and syllable durations (Chobert et al., 2014), detection of pitch variations in speech (Moreno et al., 2009), speech segmentation skills (François and Schön, 2011), and discrimination of consonants (Kraus et al., 2014b). Moreover, an association between brain responses to syllables (using the complex Auditory Brainstem Response method) and degree of active engagement (i.e., better classroom participation and attendance) in a music program suggests that the amount of training and level of engagement is an important factor in music-training-driven plasticity (Kraus et al., 2014a).

Another important aspect of the neurodevelopmental framework, thus far not definitively investigated in the literature, is that individual differences in innate (or pre-existing) musical traits may differentially affect music-training-driven plasticity and transfer to language skills. The extant literature does suggest that the relationship between language and music skills varies with different levels of music aptitude (Banai and Ahissar, 2013) and that pre-existing genetic differences likely account for some variation in level of music achievement attained (reviewed in Schellenberg, 2015). Given that individual differences in music abilities can predict some aspects of linguistic competence, even in non-musician children (Strait et al., 2011; Woodruff Carr et al., 2014; Gordon et al., 2015b), taking these individual differences into account could potentially provide a significant path to predicting response to music intervention. In this vein, Seither-Preisler et al. (2014) propose a fascinating neurocognitive model of competence development that would account for the interaction between pre-dispositions and intervention efficacy by modeling plasticity and anatomical influences on music development. They found that the size of the right Heschl's Gyrus significantly predicted variance in the amount of time that children spent practicing their instruments; the authors interpret this finding as evidence that this particular neurophysiological morphology interacts with motivational factors that determine the amount of time/effort devoted to music. More generally, it is theoretically conceivable that a subset of children has a particular brain architecture that pre-disposes them to faster musical growth and more efficient transfer to language skills; while others may have neural substrates that respond better to other types of language interventions (e.g., phonological only). Continued investigation of these and other hypotheses regarding individual differences may turn out to reduce heterogeneity of findings in future individual studies and meta-analyses on the topic of music-training-driven changes in neural and cognitive activity.

The mixed results obtained in the current meta-analysis could instead signify possible limitations of music training for literacy skills in children. Such an interpretation could be regarded in accordance with previous accounts of modularity of some aspects of language and music (Peretz, 2006). For instance, Peretz et al. (2015) argue that studies showing “neural overlap” of music and language in brain areas do not necessarily indicate that the same neuronal populations within a given brain area are active for both musical and speech processing. Moreover, it is important to bear in mind that small or non-robust effects of transfer from training to another skill are not unusual in the context of the larger literature on skills transfer. Many of the same methodological challenges (i.e., control group selection) encountered in the current meta-analysis are cited as prevalent issues for the skill-learning field much beyond music and language (Green et al., 2014). To this point, Green and Bavelier (2008) state “in the field of skill learning, transfer of learning from the trained task to even other very similar tasks is generally the exception rather than the rule.” Bransford and Schwartz (1999) suggest that the difficulty in finding consistent results of skills transfer stems in part from the idea that assessments of current knowledge generally do not capture the dynamics of the learning process. In the current meta-analysis, evidence that music training (that in some cases involves rhyming materials) has impacted performance on a standardized test of pre-reading skills (that has different surface features, cues, and demand characteristics) has crossed a substantial hurdle in establishing skills transfer; thus, even small gains should not be considered trivial.

To develop a full picture of the extent of transfer from music experiences to language skills and the possible applicability of the neuro-developmental framework, more work is also needed on the underlying mechanisms of music-related improvements in language when they are reported (either in individual studies or future meta-analyses). These effects could potentially be due to all-around, general acoustic perception/auditory processing skills (affecting perception of pitch, timing, and spectral characteristics); or, the benefits may be only specific to certain aspects of phonology such as fine-tuned detection of voice-onset-time (Zuk et al., 2013b), or perception of prosodic patterns on the supra-syllabic level. Indeed, a growing number of studies have linked speech rhythm sensitivity to early literacy skills. Sensitivity to stress patterns in spoken language are correlated with emerging reading skills in early readers (ages 5–7; Holliman et al., 2008; Goswami et al., 2010), and predict later reading development (Holliman et al., 2010). Struggling readers are also more likely to show weaknesses in perception of speech rhythm (Holliman et al., 2012) and musical rhythm (Huss et al., 2011; Flaugnacco et al., 2014). The temporal sampling theory (Goswami, 2011), along with work on neural oscillations involved in speech comprehension (Luo and Poeppel, 2007; Abrams et al., 2008: Hickok, 2012) converge in their explanation of a temporal scaffolding created by low-frequency stress patterns that facilitates acquisition and comprehension of higher-frequency (e.g., phonetic) information in the speech signal. These mechanisms may be shared by musical rhythm skills (Gordon et al., 2011; Hausen et al., 2013; Hickok et al., 2015; Morillon and Schroeder, 2015). Recent work translating related concepts of rhythm entrainment from dynamic attending theory to speech perception (Schön and Tillmann, 2015) suggest that even short-term rhythmic stimulation can impact phonological processing. A general deficit in these mechanisms of rhythm sensitivity could hinder acquisition of language and literacy skills (e.g., Leong et al., 2011; Power et al., 2013); individual differences in rhythm sensitivity could possibly mediate response to treatment, and should be taken into account. Likewise, the role of auditory working memory in music-training-driven plasticity is not yet well-understood (Kraus et al., 2012; Ramachandra et al., 2012; Tierney and Kraus, 2013b) and should be accounted for in future intervention studies. Table 5 summarizes potential questions to be addressed in future work.

The present meta-analysis contributes to the literature by examining the influence of music training on reading-related skills while also constraining the amount of reading instruction received across groups and modeling potentially important moderators (age, hours of training and type of control intervention). The findings yielded modest gains in phonological awareness (mainly in rhyming skills) for music vs. control interventions, but the small subset of studies examining reading fluency skills found no significant aggregate improvements in music vs. control groups. The literature review synthesized results from previous work suggesting potential benefits of music training on non-musical academic skills (e.g., Patel, 2011), supported by some evidence for a transfer from music training to rhyming and phonological awareness skills yielded by the present meta-analysis. This approach has also laid some groundwork for exploring specific aspects of the relationship between reading and music, which may take place in part through enhancement to perception of rhyming. This finding converges with the hypothesis that music supports phonological awareness; further study is needed to determine if intensive and long-term music training can enhance reading fluency via improvements to auditory skills, phonological awareness, and rhyming in particular. Given the limitations discussed here of the work included in this meta-analysis and the potential factors to address (summarized in Table 5), further investigation of a positive transfer from music education to reading-related skills is warranted. These investigations should eventually be considered in light of current trends in educational policy to cut funding for arts education (Kratus, 2007), such as when music lessons are eliminated in order to increase instructional time and resources for core subjects.

To draw definitive conclusions on a causal link from music to literacy and possible mediating mechanisms, there is abundant room for further progress in using longitudinal studies to address both the study design factors and the potential moderators of music-training-driven plasticity in reading-related skills. Brain imaging methods may reveal mechanisms underlying this plasticity, and can potentially be exploited to establish innovative approaches for predicting individual differences in response to music training. Recent work linking rhythmic processing to speech sound sensitivity and literacy skills suggests candidate mechanisms for improving reading skills via music education, and warrant further investigation in the context of using music training to remediate reading disabilities in school-age children. Future longitudinal studies incorporating both behavioral reading-related outcomes and measures of neural plasticity in typically developing and struggling readers are also needed in order to assess the viability of the neuro-developmental framework for music interventions.

## Conflict of interest statement

The authors declare that the research was conducted in the absence of any commercial or financial relationships that could be construed as a potential conflict of interest.

## References

[B1] Abrams D. A. Nicol T. Zecker S. Kraus N. (2008). Right-hemisphere auditory cortex is dominant for coding syllable patterns in speech. J. Neurosci. 28, 3958–3965. 10.1523/JNEUROSCI.0187-08.2008 18400895 PMC2713056

[B2] Anvari S. H. Trainor L. J. Woodside J. Levy B. A. (2002). Relations among musical skills, phonological processing, and early reading ability in preschool children. J. Exp. Child Psychol. 83, 111–130. 10.1016/S0022-0965(02)00124-8 12408958

[B3] Armand F. Montésinos-Gelet I. (2001). Apprentissage de la Lecture et de L'écriture en Milieux Pluriethniques: Études des Contextes Langagiers et du Degré D'automatisation des Processus en Lecture (Organisme subventionnaire: Immigration et métropoles). Software programmer: Michel Bastien.

[B4] Banai K. Ahissar M. (2013). Musical experience, auditory perception and reading-related skills in children. PLoS ONE 8:e75876. 10.1371/journal.pone.0075876 24086654 PMC3782483

[B5] Bhide A. Power A. Goswami U. (2013). A rhythmic musical intervention for poor readers: a comparison of efficacy with a letter-based intervention. Mind Brain Educ. 7, 113–123. 10.1111/mbe.12016

[B6] Bidelman G. M. Alain C. (2015). Musical training orchestrates coordinated neuroplasticity in auditory brainstem and cortex to counteract age-related declines in categorical vowel perception. J. Neurosci. 35, 1240–1249. 10.1523/JNEUROSCI.3292-14.2015 25609638 PMC6605547

[B7] Bidelman G. M. Weiss M. W. Moreno S. Alain C. (2014). Coordinated plasticity in brainstem and auditory cortex contributes to enhanced categorical speech perception in musicians. Eur. J. Neurosci. 40, 2662–2673. 10.1111/ejn.12627 24890664

[B8] Bolduc J. (2009). Effects of a music programme on kindergartners' phonological awareness skills. Int. J. Music Educ. 27, 37–47. 10.1177/0255761408099063

[B9] ^*^ Bolduc J. Lefebvre P. (2012). Using nursery rhymes to foster phonological and musical processing skills in kindergarteners. Creative Educ. 3, 495–502. 10.4236/ce.2012.34075

[B10] Bransford J. D. Schwartz D. L. (1999). Rethinking transfer: a simple proposal with multiple implications. Rev. Res. Educ. 24, 61–100.

[B11] Brod G. Opitz B. (2012). Does it really matter? Separating the effects of musical training on syntax acquisition. Front. Psychol. 3:543. 10.3389/fpsyg.2012.00543 23248608 PMC3521129

[B12] Bus A. G. van IJzendoorn M. H. (1999). Phonological awareness and early reading: a meta-analysis of experimental training studies. J. Educ. Psychol. 91:403 10.1037/0022-0663.91.3.403

[B13] Butera I. M. (2015). From notes to vowels: neural correlations between musical training and speech processing. J. Neurosci. 35, 8379–8381. 10.1523/JNEUROSCI.1102-15.2015 26041906 PMC4452548

[B14] Butzlaff R. (2000). Can music be used to teach reading? J. Aesthetic Educ. 34, 167–178. 10.2307/3333642

[B15] Capovilla A. Capovilla F. (1998). Prova de conscie^ncia fonolo ´gica: desenvolvi- mento de dez habilidades da pre ´-escola a ´segunda se ´rie [Phonological Awareness Test: development of tem abilities from presschool to third grade]. Temas Desenvol. 7, 14–20.

[B16] Chobert J. Francois C. Velay J. L. Besson M. (2014). Twelve months of active musical training in 8- to 10-year-old children enhances the preattentive processing of syllabic duration and voice onset time. Cereb. Cortex 24, 956–967. 10.1093/cercor/bhs377 23236208

[B17] Chobert J. Marie C. Francois C. Schön D. Besson M. (2011). Enhanced passive and active processing of syllables in musician children. J. Cogn. Neurosci. 23, 3874–3887. 10.1162/jocn_a_00088 21736456

[B18] ^*^ Cogo-Moreira H. Brandão de Ávila C. R. Ploubidis G. B. Mari J. D. J. (2013). Effectiveness of music education for the improvement of reading skills and academic achievement in young poor readers: a pragmatic cluster-randomized, controlled clinical trial. PLoS ONE 8:e59984. 10.1371/journal.pone.0059984 23544117 PMC3609825

[B19] Darrow A.-A. (2009). Enhancing literacy in the second grade: five related studies using the register music/reading curriculum. Appl. Res. Music Educ. 27, 12–26. 10.1177/8755123308330044

[B20] ^*^ Degé F. Schwarzer G. (2011). The effect of a music program on phonological awareness in preschoolers. Front. Psychol. 2:124. 10.3389/fpsyg.2011.00124 21734895 PMC3121007

[B21] Dellatolas G. Watier L. Le Normand M. T. Lubart T. Chevrie-Muller C. (2009). Rhythm reproduction in kindergarten, reading performance at second grade, and developmental dyslexia theories. Arch. Clin. Neuropsychol. 24, 555–563. 10.1093/arclin/acp044 19628461

[B22] Dickinson D. Chaney C. (1997). Early Phonological Awareness Profile. Newton, MA: Education Development Center.

[B23] Douglas S. Willatts P. (1994). The relationship between musical ability and literacy skills. J. Res. Read. 17, 99–107. 10.1111/j.1467-9817.1994.tb00057.x

[B24] Evans S. Meekings S. Nuttall H. E. Jasmin K. M. Boebinger D. Adank P.. (2014). Does musical enrichment enhance the neural coding of syllables? Neuroscientific interventions and the importance of behavioral data. Front. Hum. Neurosci. 8:964. 10.3389/fnhum.2014.00964 25566013 PMC4267267

[B25] Flaugnacco E. Lopez L. Terribili C. Zoia S. Buda S. Tilli S.. (2014). Rhythm perception and production predict reading abilities in developmental dyslexia. Front. Hum. Neurosci. 8:392. 10.3389/fnhum.2014.00392 24926248 PMC4045153

[B26] Forgeard M. Schlaug G. Norton A. Rosam C. Iyangar U. (2008). The relations between music and phonological processing in normal-reading children and children with dyslexia. Music Percept. 25, 383–390. 10.1525/mp.2008.25.4.383

[B27] François C. Schön D. (2011). Musical expertise boosts implicit learning of both musical and linguistic structures. Cereb. Cortex 21, 2357–2365. 10.1093/cercor/bhr022 21383236

[B28] Frederickson N. Frith U. Reason R. (1997). Phonological Assessment Battery: Standardised Edition. Windsor: NFER-Nelson.

[B29] Good R. Kaminski R. (2001). Dynamic Indicators of Basic Early Literacy Skills, 5th Edn. Eugene, OR: Institute for the Development of Educational Achievement.

[B30] Good R. Kaminski R. (2002). Dynamic Indicators of Basic Early Literacy Skills. Eugene, OR: Institute for the Development of Educational Achievement.

[B31] Gordon R. L. Jacobs M. S. Schuele C. M. McAuley J. D. (2015a). Perspectives on the rhythm-grammar link and its implications for typical and atypical language development. Ann. N.Y. Acad. Sci. 1337, 16–25. 10.1111/nyas.12683 25773612 PMC4794983

[B32] Gordon R. L. Magne C. L. Large E. W. (2011). EEG correlates of song prosody: a new look at the relationship between linguistic and musical rhythm. Front. Psychol. 2:352. 10.3389/fpsyg.2011.00352 22144972 PMC3225926

[B33] Gordon R. L. Shivers C. M. Wieland E. A. Kotz S. A. Yoder P. J. Devin McAuley J. (2015b). Musical rhythm discrimination explains individual differences in grammar skills in children. Dev. Sci. 18, 635–644. 10.1111/desc.12230 25195623

[B34] Goswami U. (2011). A temporal sampling framework for developmental dyslexia. Trends Cogn. Sci. (Regul. Ed). 15, 3–10. 10.1016/j.tics.2010.10.001 21093350

[B35] Goswami U. Gerson D. Astruc L. (2010). Amplitude envelope perception, phonology and prosodic sensitivity in children with developmental dyslexia. Read. Writ. 23, 995–1019. 10.1007/s11145-009-9186-6

[B36] Goswami U. Huss M. Mead N. Fosker T. Verney J. P. (2013). Perception of patterns of musical beat distribution in phonological developmental dyslexia: significant longitudinal relations with word reading and reading comprehension. Cortex 49, 1363–1376. 10.1016/j.cortex.2012.05.005 22726605

[B37] Green C. S. Bavelier D. (2008). Exercising your brain: a review of human brain plasticity and training-induced learning. Psychol. Aging 23, 692–701. 10.1037/a0014345 19140641 PMC2896818

[B38] Green C. S. Strobach T. Schubert T. (2014). On methodological standards in training and transfer experiments. Psychol. Res. 78, 756–772. 10.1007/s00426-013-0535-3 24346424

[B39] ^*^ Gromko J. E. (2005). The effect of music instruction on phonemic awareness in beginning readers. J. Res. Music Educ. 53, 199–209. 10.1177/002242940505300302

[B40] Hambrick D. Z. Oswald F. L. Altmann E. M. Meinz E. J. Gobet F. Campitelli G. (2014). Deliberate practice: is that all it takes to become an expert? Intelligence 45, 34–45. 10.1016/j.intell.2013.04.001

[B41] Harris P. A. Taylor R. Thielke R. Payne J. Gonzalez N. Conde J. G. (2009). Research electronic data capture (REDCap)–a metadata-driven methodology and workflow process for providing translational research informatics support. J. Biomed. Inform. 42, 377–381. 10.1016/j.jbi.2008.08.010 18929686 PMC2700030

[B42] Hausen M. Torppa R. Salmela V. R. Vainio M. Särkämö T. (2013). Music and speech prosody: a common rhythm. Front. Psychol. 4:566. 10.3389/fpsyg.2013.00566 24032022 PMC3759063

[B43] ^*^ Herrera L. Lorenzo O. Defior S. Fernandez-Smith G. Costa-Giomi E. (2011). Effects of phonological and musical training on the reading readiness of native- and foreign-Spanish-speaking children. Psychol. Music 39, 68–81. 10.1177/0305735610361995

[B44] Hickok G. (2012). Computational neuroanatomy of speech production. Nat. Rev. Neurosci. 13, 135–145. 10.1038/nrn3158 22218206 PMC5367153

[B45] Hickok G. Farahbod H. Saberi K. (2015). The rhythm of perception: entrainment to acoustic rhythms induces subsequent perceptual oscillation. Psychol. Sci. 26, 1006–1013. 10.1177/0956797615576533 25968248 PMC4504793

[B46] Higgins J. P. Thompson S. G. (2002). Quantifying heterogeneity in a meta-analysis. Stat. Med. 21, 1539–1558. 10.1002/sim.1186 12111919

[B47] Holliman A. J. Wood C. Sheehy K. (2008). Sensitivity to speech rhythm explains individual differences in reading ability independently of phonological awareness. Br. J. Dev. Psychol. 26, 357–367. 10.1348/026151007X241623

[B48] Holliman A. J. Wood C. Sheehy K. (2010). Does speech rhythm sensitivity predict children's reading ability 1 year later? J. Educ. Psychol. 102, 356–366. 10.1037/a0018049

[B49] Holliman A. J. Wood C. Sheehy K. (2012). A cross-sectional study of prosodic sensitivity and reading difficulties. J. Res. Read. 35, 32–48. 10.1111/j.1467-9817.2010.01459.x

[B50] Huss M. Verney J. P. Fosker T. Mead N. Goswami U. (2011). Music, rhythm, rise time perception and developmental dyslexia: perception of musical meter predicts reading and phonology. Cortex 47, 674–689. 10.1016/j.cortex.2010.07.010 20843509

[B51] Hyde K. L. Lerch J. Norton A. Forgeard M. Winner E. Evans A. C.. (2009). Musical training shapes structural brain development. J. Neurosci. 29, 3019–3025. 10.1523/JNEUROSCI.5118-08.2009 19279238 PMC2996392

[B52] Jansen H. Mannhaupt G. Marx H. Skowronek H. (2002). Bielefelder Screening zur Früherkennung von Lese-Rechtschreibschwierigkeiten. Göttingen: Hogrefe.

[B53] Kratus J. (2007). Music education at the tipping point. Music Educ. J. 94, 42–48. 10.1177/002743210709400209

[B54] Kraus N. Chandrasekaran B. (2010). Music training for the development of auditory skills. Nat. Rev. Neurosci. 11, 599–605. 10.1038/nrn2882 20648064

[B55] Kraus N. Slater J. Thompson E. C. Hornickel J. Strait D. L. Nicol T.. (2014a). Auditory learning through active engagement with sound: biological impact of community music lessons in at-risk children. Front. Neurosci. 8:351. 10.3389/fnins.2014.00351 25414631 PMC4220673

[B56] Kraus N. Slater J. Thompson E. C. Hornickel J. Strait D. L. Nicol T.. (2014b). Music enrichment programs improve the neural encoding of speech in at-risk children. J. Neurosci. 34, 11913–11918. 10.1523/JNEUROSCI.1881-14.2014 25186739 PMC6608462

[B57] Kraus N. Strait D. L. Parbery-Clark A. (2012). Cognitive factors shape brain networks for auditory skills: spotlight on auditory working memory. Ann. N.Y. Acad. Sci. 1252, 100–107. 10.1111/j.1749-6632.2012.06463.x 22524346 PMC3338202

[B58] Lamb S. J. Gregory A. H. (1993). The relationship between music and reading in beginning readers. Educ. Psychol. 13, 19–27. 10.1080/0144341930130103

[B59] Leong V. Goswami U. (2014). Assessment of rhythmic entrainment at multiple timescales in dyslexia: evidence for disruption to syllable timing. Hear. Res. 308, 141–161. 10.1016/j.heares.2013.07.015 23916752 PMC3969307

[B60] Leong V. Hamalainen J. Soltesz F. Goswami U. (2011). Rise time perception and detection of syllable stress in adults with developmental dyslexia. J. Mem. Lang. 64, 59–73. 10.1016/j.jml.2010.09.003

[B61] Lipsey M. W. Wilson D. B. (2001). Practical Meta-analysis. Thousand Oaks, CA: Sage Publications.

[B62] Lonigan C. J. Shanahan T. (2009). Developing Early Literacy: Report of the National Early Literacy Panel. Executive Summary. A Scientific Synthesis of Early Literacy Development and Implications for Intervention. Washington, DC: National Institute for Literacy.

[B63] Luo H. Poeppel D. (2007). Phase patterns of neuronal responses reliably discriminate speech in human auditory cortex. Neuron 54, 1001–1010. 10.1016/j.neuron.2007.06.004 17582338 PMC2703451

[B64] Magne C. Schön D. Besson M. (2006). Musician children detect pitch violations in both music and language better than nonmusician children: behavioral and electrophysiological approaches. J. Cogn. Neurosci. 18, 199–211. 10.1162/jocn.2006.18.2.199 16494681

[B65] Marie C. Magne C. Besson M. (2011). Musicians and the metric structure of words. J. Cogn. Neurosci. 23, 294–305. 10.1162/jocn.2010.21413 20044890

[B66] Melby-Lervag M. Lyster S. A. Hulme C. (2012). Phonological skills and their role in learning to read: a meta-analytic review. Psychol. Bull. 138, 322–352. 10.1037/a0026744 22250824

[B67] Milovanov R. Huotilainen M. Esquef P. A. Alku P. Välim4¨ki V. Tervaniemi M. (2009). The role of musical aptitude and language skills in preattentive duration processing in school-aged children. Neurosci. Lett. 460, 161–165. 10.1016/j.neulet.2009.05.063 19481587

[B68] ^*^ Moreno S. Friesen D. Bialystok E. (2011). Effect of music training on promoting preliteracy skills: preliminary causal evidence. Music Percept. 29, 165–172. 10.1525/mp.2011.29.2.165

[B69] ^*^ Moreno S. Marques C. Santos A. Santos M. Castro S. L. Besson M. (2009). Musical training influences linguistic abilities in 8-year-old children: more evidence for brain plasticity. Cereb. Cortex 19, 712–723. 10.1093/cercor/bhn120 18832336

[B70] Morillon B. Schroeder C. E. (2015). Neuronal oscillations as a mechanistic substrate of auditory temporal prediction. Ann. N.Y. Acad. Sci. 1337, 26–31. 10.1111/nyas.12629 25773613 PMC4363099

[B71] ^*^ Moritz C. Yampolsky S. Papadelis G. Thomson J. Wolf M. (2013). Links between early rhythm skills, musical training, and phonological awareness. Read. Writ. 26, 739–769. 10.1007/s11145-012-9389-0

[B72] ^*^ Myant M. Armstrong W. Healy N. (2008). Can music make a difference? A small scale longitudinal study into the effects of music instruction in nursery on later reading ability. Educ. Child Psychol. 25, 83–100.

[B73] Overy K. (2003). Dyslexia and music: from timing deficits to musical intervention. Ann. N. Y. Acad. Sci. 999, 497–505. 10.1196/annals.1284.060 14681173

[B74] Parbery-Clark A. Anderson S. Kraus N. (2013). Musicians change their tune: how hearing loss alters the neural code. Hear. Res. 302, 121–131. 10.1016/j.heares.2013.03.009 23566981

[B75] Parbery-Clark A. Strait D. L. Anderson S. Hittner E. Kraus N. (2011). Musical experience and the aging auditory system: implications for cognitive abilities and hearing speech in noise. PLoS ONE 6:e18082. 10.1371/journal.pone.0018082 21589653 PMC3092743

[B76] Patel A. D. (2008). Music, Language, and the Brain. New York, NY: Oxford University Press.

[B77] Patel A. D. (2011). Why would musical training benefit the neural encoding of speech? The OPERA hypothesis. Front. Psychol. 2:142. 10.3389/fpsyg.2011.00142 21747773 PMC3128244

[B78] Patel A. D. (2014). Can nonlinguistic musical training change the way the brain processes speech? The expanded OPERA hypothesis. Hear. Res. 308, 98–108. 10.1016/j.heares.2013.08.011 24055761

[B79] Peretz I. (2006). The nature of music from a biological perspective. Cognition 100, 1–32. 10.1016/j.cognition.2005.11.004 16487953

[B80] Peretz I. Vuvan D. Lagrois M. É. Armony J. L. (2015). Neural overlap in processing music and speech. Philos. Trans. R. Soc. Lond. B Biol. Sci. 370:20140090. 10.1098/rstb.2014.0090 25646513 PMC4321131

[B81] Peynircioglu Z. F. Durgunoglu A. Y. Úney-Küsefogˇlu B. (2002). Phonological awareness and musical aptitude. J. Res. Read. 25, 68–80. 10.1111/1467-9817.00159

[B82] Power A. J. Mead N. Barnes L. Goswami U. (2013). Neural entrainment to rhythmic speech in children with developmental dyslexia. Front. Hum. Neurosci. 7:777. 10.3389/fnhum.2013.00777 24376407 PMC3842021

[B83] Ramachandra V. Meighan C. Gradzki J. (2012). The impact of musical training on the phonological memory and the central executive: a brief report. N. Am. J. Psychol. 14, 541–548.

[B84] R Core Team (2015). R: A Language and Environment for Statistical Computing. Vienna: R Core Team.

[B85] Register D. (2001). The effects of an early intervention music curriculum on prereading/writing. J. Music Ther. 38, 239–248. 10.1093/jmt/38.3.239 11570934

[B86] ^*^ Register D. (2004). The effects of live music groups versus an educational children's television program on the emergent literacy of young children. J. Music Ther. 41, 2–27. 10.1093/jmt/41.1.2 15157126

[B87] Register D. Darrow A.-A. Standley J. Swedberg O. (2007). The use of music to enhance reading skills of second grade students and students with reading disabilities. J. Music Ther. 44, 23–37. 10.1093/jmt/44.1.23 17419662

[B88] Robertson C. Salter W. (1997). The Phonological Awareness Test. East Moline, IL: LinguiSystems.

[B89] Schellenberg E. G. (2015). Music training and speech perception: a gene-environment interaction. Ann. N.Y. Acad. Sci. 1337, 170–177. 10.1111/nyas.12627 25773632

[B90] Schlaggar B. L. McCandliss B. D. (2007). Development of neural systems for reading. Annu. Rev. Neurosci. 30, 475–503. 10.1146/annurev.neuro.28.061604.135645 17600524

[B91] Schön D. Tillmann B. (2015). Short- and long-term rhythmic interventions: perspectives for language rehabilitation. Ann. N.Y. Acad. Sci. 1337, 32–39. 10.1111/nyas.12635 25773614

[B92] Seither-Preisler A. Parncutt R. Schneider P. (2014). Size and synchronization of auditory cortex promotes musical, literacy, and attentional skills in children. J. Neurosci. 34, 10937–10949. 10.1523/JNEUROSCI.5315-13.2014 25122894 PMC6705250

[B93] Shahin A. Bosnyak D. J. Trainor L. J. Roberts L. E. (2003). Enhancement of neuroplastic P2 and N1c auditory evoked potentials in musicians. J. Neurosci. 23, 5545–5552. 12843255 10.1523/JNEUROSCI.23-13-05545.2003 PMC6741225

[B94] Skoe E. Kraus N. (2012). A little goes a long way: how the adult brain is shaped by musical training in childhood. J. Neurosci. 32, 11507–11510. 10.1523/JNEUROSCI.1949-12.2012 22915097 PMC6703757

[B95] Slevc L. R. Miyake A. (2006). Individual differences in second-language proficiency: does musical ability matter? Psychol. Sci. 17, 675–681. 10.1111/j.1467-9280.2006.01765.x 16913949

[B96] Standley J. M. (2008). Does music instruction help children learn to read? Evidence of a meta-analysis. Appl. Res. Music Educ. 27, 17–32. 10.1177/8755123308322270

[B97] Standley J. M. Hughes J. E. (1997). Evaluation of an early intervention music curriculum for enhancing prereading/writing skills. Music Ther. Perspect. 15, 79–85. 10.1093/mtp/15.2.79

[B98] Strait D. L. Hornickel J. Kraus N. (2011). Subcortical processing of speech regularities underlies reading and music aptitude in children. Behav. Brain Funct. 7:44. 10.1186/1744-9081-7-44 22005291 PMC3233514

[B99] Succena A. Castro S. (2010). Bateria de Avaliaçao da Leitura em Português Europeu. Lisboa: CEGOC.

[B100] ^*^ Thomson J. M. Leong V. Goswami U. (2013). Auditory processing interventions and developmental dyslexia: a comparison of phonemic and rhythmic approaches. Read. Writ. 26, 139–161. 10.1007/s11145-012-9359-6

[B101] Tierney A. Kraus N. (2013a). Music training for the development of reading skills. Prog. Brain Res. 207, 209–241. 10.1016/B978-0-444-63327-9.00008-4 24309256

[B102] Tierney A. T. Kraus N. (2013b). The ability to tap to a beat relates to cognitive, linguistic, and perceptual skills. Brain Lang. 124, 225–231. 10.1016/j.bandl.2012.12.014 23400117 PMC3594434

[B103] Torgesen J. Wagner R. Rashotte C. (1999). Test of Word Reading Efficiency (TOWRE). Austin, TX: ProEd.

[B104] Viechtbauer W. (2010). Conducting meta-analyses in R with the metafor package. J. Stat. Softw. 36, 1–48. 10.18637/jss.v036.i03

[B105] Walker K. M. Hall S. E. Klein R. M. Phillips D. P. (2006). Development of perceptual correlates of reading performance. Brain Res. 1124, 126–141. 10.1016/j.brainres.2006.09.080 17069776

[B106] Wong P. C. Skoe E. Russo N. M. Dees T. Kraus N. (2007). Musical experience shapes human brainstem encoding of linguistic pitch patterns. Nat. Neurosci. 10, 420–422. 10.1038/nn1872 17351633 PMC4508274

[B107] Woodcock R. W. Mather N. McGrew K. S. (2001). Woodcock-Johnson III Tests of Cognitive Abilities Examiner's Manual. Itasca, IL: Riverside

[B108] Woodruff Carr K. White-Schwoch T. Tierney A. T. Strait D. L. Kraus N. (2014). Beat synchronization predicts neural speech encoding and reading readiness in preschoolers. Proc. Natl. Acad. Sci. U.S.A. 111, 14559–14564. 10.1073/pnas.1406219111 25246562 PMC4210020

[B109] ^*^ Yazejian N. Peisner-Feinberg E. S. (2009). Effects of a preschool music and movement curriculum on children's language skills. NHSA Dialog. 12, 327–341. 10.1080/15240750903075255

[B110] Zuk J. Andrade P. E. Andrade O. V. Gardiner M. Gaab N. (2013a). Musical, language, and reading abilities in early Portuguese readers. Front. Psychol. 4:288. 10.3389/fpsyg.2013.00288 23785339 PMC3684766

[B111] Zuk J. Ozernov-Palchik O. Kim H. Lakshminarayanan K. Gabrieli J. D. Tallal P.. (2013b). Enhanced syllable discrimination thresholds in musicians. PLoS ONE 8:e80546. 10.1371/journal.pone.0080546 24339875 PMC3855080

