# Supplementary material for: Does Music Training Enhance Literacy Skills? A Meta-Analysis
Source: Front Psychol. 2015 Dec 1;6:1777. doi: 10.3389/fpsyg.2015.01777 (PMC4664655; doi:10.3389/fpsyg.2015.01777)
Supplement: Supplementary file 1 [file Table1.DOCX]

Supplementary Material

Does music training enhance literacy skills? A meta-analysis

Reyna L. Gordon*, Hilda M. Fehd, Bruce D. McCandliss

*** Correspondence:** reyna.gordon@vanderbilt.edu

**Supplementary Table 1. Search terms used for each of the databases.**

| **Database** | **Search Terms** |
| --- | --- |
| Proquest (with the following databases selected):   - ERIC - International Index to Music Periodicals Full Text - Linguistics and Language Behavior Abstracts - MLA International Bibliography - ProQuest Education Journals - ProQuest Psychology Journals - ProQuest Research Library - ProQuest Science Journals - ProQuest Social Science Journals - PsychARTICLES - PsycINFO - RILM Abstracts of Music Literature | = all(brain PRE/1 development OR auditory PRE/1 processing OR phon* PRE/1 awareness OR reading PRE/1 abilit* OR reading PRE/1 outcome* OR reading PRE/1 fluency OR reading PRE/1 achievement OR reading PRE/1 skill* OR reading PRE/1 readiness OR *literacy OR prereading OR linguistic PRE/1 abilit* OR academic PRE/1 achievement OR phon* OR verbal OR phon* PRE/1 decoding OR decoding PRE/1 skill*) AND all(music PRE/1 lesson* OR music* PRE/1 training OR music PRE/1 curriculum OR music PRE/1 education OR music* PRE/1 intervention OR music* PRE/1 instruction OR music* PRE/1 approach* OR rhythmic* PRE/1 approach* OR music* PRE/1 experience OR music* PRE/1 program OR music* PRE/1 group*) |
| ISI Web of Knowledge | = ("brain development" OR "auditory processing" OR "phon* awareness" OR "reading abilit*" OR "reading outcome*" OR "reading fluency" OR "reading achievement" OR "reading skill*" OR "reading readiness" OR "*literacy" OR "prereading" OR "linguistic abilit*" OR "academic achievement" OR "phon*" OR "verbal" OR "phon* decoding" OR "decoding skill*") AND ("music lesson*" OR "music* training" OR "music curriculum" OR "music education" OR "music* intervention" OR "music* instruction" OR "music* approach*" OR "rhythmic* approach*" OR "music* experience" OR "music* program" OR "music* group*") |
| PubMed | = ((brain development) OR (auditory processing) OR (phon* awareness) OR (reading abilit*) OR (reading outcome*) OR (reading fluency) OR (reading achievement) OR (reading skill*) OR (reading readiness) OR (*literacy) OR (prereading) OR (linguistic abilit*) OR (academic achievement) OR (phon*) OR (verbal) OR (phon* decoding) OR (decoding skill*) OR (language) OR (phon* skill*)) AND ((music lesson*) OR (music* training) OR (music curriculum) OR (music education) OR (music* intervention) OR (music* instruction) OR (music* approach*) OR (rhythmic* approach*) OR (music* experience) OR (music* program) OR (music* group*)) |
